# Role of Integrin αvβ3 in Doxycycline-Induced Anti-Proliferation in Breast Cancer Cells

**DOI:** 10.3389/fcell.2022.829788

**Published:** 2022-02-14

**Authors:** Yi-Fong Chen, Yung-Ning Yang, Hung-Ru Chu, Tung-Yung Huang, Shwu-Huey Wang, Han-Yu Chen, Zi-Lin Li, Yu-Chen S. H. Yang, Hung-Yun Lin, Aleck Hercbergs, Jacqueline Whang-Peng, Kuan Wang, Paul J. Davis

**Affiliations:** ^1^ Graduate Institute of Cancer Biology and Drug Discovery, College of Medical Science and Technology, Taipei Medical University, Taipei, Taiwan; ^2^ Graduate Institute of Nanomedicine and Medical Engineering, College of Medical Engineering, Taipei Medical University, Taipei, Taiwan; ^3^ School of Medicine, I-Shou University, Kaohsiung, Taiwan; ^4^ Department of Pediatrics, E-DA Hospital, Kaohsiung, Taiwan; ^5^ Core Facility Center, Department of Research Development, Taipei Medical University, Taipei, Taiwan; ^6^ Joint Biobank, Office of Human Research, Taipei Medical University, Taipei, Taiwan; ^7^ Cancer Center, Wan Fang Hospital, Taipei Medical University, Taipei, Taiwan; ^8^ TMU Research Center of Cancer Translational Medicine, Taipei Medical University, Taipei, Taiwan; ^9^ Traditional Herbal Medicine Research Center of Taipei Medical University Hospital, Taipei Medical University, Taipei, Taiwan; ^10^ Pharmaceutical Research Institute, Albany College of Pharmacy and Health Sciences, Albany, NY, United States; ^11^ Department of Radiation Oncology, Cleveland Clinic, Cleveland, OH, United States; ^12^ Department of Medicine, Albany Medical College, Albany, NY, United States

**Keywords:** doxycycline, anti-proliferation, integrin αvβ3, signal transduction, breast cancer

## Abstract

Doxycycline, an antibiotic, displays the inhibition of different signal transduction pathways, such as anti-inflammation and anti-proliferation, in different types of cancers. However, the anti-cancer mechanisms of doxycycline *via* integrin αvβ3 are incompletely understood. Integrin αvβ3 is a cell-surface anchor protein. It is the target for estrogen, androgen, and thyroid hormone and plays a pivotal role in the proliferation, migration, and angiogenic process in cancer cells. In our previous study, thyroxine hormones can interact with integrin αvβ3 to activate the extracellular signal-regulated kinase 1/2 (ERK1/2), and upregulate programmed death-ligand 1 (*PD-L1*) expression. In the current study, we investigated the inhibitory effects of doxycycline on proliferation in two breast cancer cell lines, MCF-7 and MDA-MB-231 cells. Doxycycline induces concentration-dependent anti-proliferation in both breast cancer cell lines. It regulates gene expressions involved in proliferation, pro-apoptosis, and angiogenesis. Doxycycline suppresses cell cyclin D1 (*CCND1*) and *c-Myc* which play crucial roles in proliferation. It also inhibits *PD-L1* gene expression. Our findings show that modulation on integrin αvβ3 binding activities changed both thyroxine- and doxycycline-induced signal transductions by an integrin αvβ3 inhibitor (HSDVHK-NH_2_). Doxycycline activates phosphorylation of focal adhesion kinase (FAK), a downstream of integrin, but inhibits the ERK1/2 phosphorylation. Regardless, doxycycline-induced FAK phosphorylation is blocked by HSDVHK-NH_2_. In addition, the specific mechanism of action associated with pERK1/2 inhibition *via* integrin αvβ3 is unknown for doxycycline treatment. On the other hand, our findings indicated that inhibiting ERK1/2 activation leads to suppression of *PD-L1* expression by doxycycline treatment. Furthermore, doxycycline-induced gene expressions are disturbed by a specific integrin αvβ3 inhibitor (HSDVHK-NH_2_) or a mitogen-activated protein kinase (MAPK)/extracellular signal-regulated kinases (ERK) kinase (MAPK/ERK, MEK) inhibitor (PD98059). The results imply that doxycycline may interact with integrin αvβ3 and inhibits ERK1/2 activation, thereby regulating cell proliferation and downregulating *PD-L1* gene expression in estrogen receptor (ER)-negative breast cancer MDA-MB-231 cells.

## Highlights

- The doxycycline inhibits proliferative genes including a cell-cycle related gene (cell cyclin D1, *CCND1*) and the oncogene (*c-Myc*) in breast cancer cells.
- The doxycycline may interact with integrin αvβ3 in ER-negative MDA-MB-231 breast cancer to down-regulate ERK1/2 activity and suppresses cancer cell growth sequentially.
- Integrin αvβ3 plays an important role in several gene expressions and antiproliferation in MDA-MB-231 cells through doxycycline-induced signal transduction.

## 1 Introduction

Doxycycline is an antibiotic for bacterial infections. However, previous studies indicate that doxycycline can promote apoptotic procedures to suppress cell growth in different kinds of cancers (Sun et al., 2009). In addition to inhibiting mitochondrial activities, doxycycline suppresses transcription of matrix metalloproteinases (MMPs), MMP-2 and MMP-9 (Wang et al., 2015). MMPs degrade matrix to induce cell migration and thus promote tumor cell spread and metastasis (Chen et al., 2005). Doxycycline suppresses lipopolysaccharide (LPS)-activated phosphorylation of p38 mitogen-activated protein kinase (MAPK) and nuclear translocation of nuclear factor kappa B (NF-κB) (Santa-Cecília et al., 2016). Furthermore, it inhibits focal adhesion kinase (FAK) phosphorylation and its transcription (Sun et al., 2009; Wang et al., 2015). FAK modulates normal cells proliferation, survival, and motility as well as in tumor cells (Hanks et al., 2003). FAK is a central signaling component triggered by numerous stimuli *via* growth factor receptors and integrins. On the other hand, doxycycline further reduces the transcription of the microglial activation marker ionized calcium binding adaptor molecule 1 (IBA-1), the production of reactive oxygen species (ROS), and nitrogen monoxide (NO) (Santa-Cecília et al., 2016). Doxycycline also reduces proinflammatory cytokines such as tumor necrosis factor alpha (TNF-α) and interleukin 1 beta (IL-1β) (Di Caprio et al., 2015). Doxycycline down-regulates vascular endothelial growth factor A (*VEGF-A*) expression which may also be connected to cancer proliferation and inflammation (Veron et al., 2012). Knockdown of VEGF disrupts integrin αvβ3 activity *via* decreasing VEGFR2 signaling. In addition, the integrin αvβ3 is a critical regulator of programmed death-ligand 1 (*PD-L1*) expression (Vannini et al., 2019). The immune checkpoint programmed cell death-1/PD-ligand 1 (PD-1/PD-L1) is associated with pro-inflammatory cytokine production and cancer growth (Huang et al., 2019). However, the link between *PD-L1* suppression *via* modulation of integrin αvβ3 and doxycycline-induced anti-proliferation of cancer cells is uncertain. Regardless, doxycycline is shown to suppress gastric cancer cell growth and the potential formation of colonies and spheroid (Pandian et al., 2020). Furthermore, a clinical trial study has shown that doxycycline can reduce cancer stem cells (CSCs) in breast cancer patients *in vivo* (Scatena et al., 2018). It is merit to investigate whether doxycycline can be used to treat breast cancer.

Integrin αvβ3 is one of 24 αβ-paired integrins that play vital roles in cell adhesion, migration, and interaction with extracellular matrix (ECM) proteins (Mezu-Ndubuisi and Maheshwari, 2021). The integrin αv subunit pairs with β1, β3, β5, β6, and β8 to form heterodimers (Luo et al., 2007). Although studies indicate that overexpressed integrin αvβ3s are observed in normal cells, they may function differently from the matter on cancer cells. Integrin αvβ3 is recognized by several binding ligands such as fibrillin, fibrinogen, fibronectin, osteopontin, tenascin, thrombospondin vitronectin, and von Willebrand factor (VWF), however, integrin αvβ5 only preferentially binds to vitronectin (Weis and Cheresh, 2011). The overexpression of integrin αvβ3 is observed in highly-growth endothelial cells and cancer cells (Cheng et al., 2021). The integrin extracellular domain controls ligand-binding specificity. On the other hand, the cytoplasmic short tail regulates intracellular signaling pathways by recruiting and activating specific kinases and signaling intermediates. Consequently, coordination between domains controls the adhesion (Altei et al., 2020), morphology (Lange et al., 2016), migration (Sales et al., 2019), invasion (Hood and Cheresh, 2002), proliferation (Moreno-Layseca and Streuli, 2014), and survival (Desgrosellier and Cheresh, 2010) in cancer cells. These signals manipulate the host microenvironment to deliver abundant blood vessel and stromal resources to maintain proliferation and metastasis of cancer cells (Luo et al., 2007). Targeting integrin αvβ3 has been interesting research for cancer prevention and targeting theragnostic therapy (Slack et al., 2022). In addition to thyroid hormone, dihydrotestosterone (DHT) and estrogen can also bind to integrin αvβ3 and thus activate biological activities (Meng et al., 2011; Ho et al., 2020). Conversely, other molecules such as resveratrol bind to the integrin αvβ3 receptor to activate anti-proliferation in different types of cancer cells (Lin et al., 2006). Additionally, thyroid hormone analogue, tetraiodothyroacetic acid (tetrac), and its nano-derivative (nano-diamino-tetrac, NDAT) not only bind to integrin αvβ3 competently with thyroid hormones but also induce anti-proliferation against cancer cells (Yang et al., 2021). The previous study reveals that doxycycline down-regulates integrin αvβ3 downstream FAK signaling in leukemia cells (Wang et al., 2015). Accordingly, those observations encourage us to investigate that the activity of doxycycline may be controlled by an integrin αvβ3-dependent signal transduction pathway.

Early studies demonstrate that doxycycline is highly lipophilic, hence it can penetrate the cell membrane and enter cells to inhibit the synthesis of 30S ribosomal subunits in bacteria (Chopra and Roberts, 2001). In the present study, we investigated the role of doxycycline-induced anti-proliferation in both estrogen receptor (ER)-positive (MCF-7) and ER-negative (MDA-MB-231) breast cancer cell lines. In contrast to the antibiotic activity of doxycycline, it inhibits the activation of extracellular signal-regulated kinase 1/2 (ERK1/2) and regulates genes that regulate cell proliferation by interacting with cell surface integrin αvβ3 in ER-negative breast cancer cells. On the other hand, thyroxine (T_4_)-induced cancer proliferation is also moderated by integrin αvβ3, which regulates ERK1/2 activation and gene expressions. Additionally, the ERK1/2 activation is associated with the *PD-L1* expression. In contrast to T_4_, the *PD-L1* gene expression is suppressed by doxycycline. The doxycycline-induced signal transductions *via* integrin αvβ3 play pivotal modulators in several gene expressions and anti-proliferation in breast cancer cells.

## 2 Materials and Methods

### 2.1 Materials

All reagents were obtained commercially and used without further purification. Thyroxine (T_4_; Cat. No. T2376) and doxycycline (Cat. No. D9891) were purchased from Sigma-Aldrich (St. Louis, MO, United States). The integrin αvβ3 inhibitor (HSDVHK-NH_2_) was purchased from Calbiochem (San Diego, CA, United States). The ERK1/2 inhibitor (PD98059; Cat. No. #9900) was purchased from Cell Signaling Technology (Danvers, MA, United States).

### 2.2 Cell Lines

Human breast cancer ER-positive MCF-7 cells and ER-negative MDA-MB-231 cells were purchased from American Type Culture Collection (ATCC) (Manassas, VA, United States ). Cell lines were tested and authenticated with BCRC (isoenzyme analysis, *Mycoplasma*, cytogenetics, tumorigenesis, receptor expression testing). Cells were cultured in Dulbecco’s Modified Eagle’s Medium (DMEM) (Life Technologies Corp., Carlsbad, CA, United States ). Media were supplemented with 10% fetal bovine serum (FBS) and 1% penicillin/streptomycin. Cell cultures were maintained in the incubator with a 5% CO_2_ supply at 37°C. Before the study, cells were placed in a 0.25% hormone-depleted serum-supplemented medium for 48 h.

### 2.3 Cell Viability Assay

MCF-7 and MDA-MB cells were plated at a density of 4 × 10^3^ cells/well in 96-well plates. Cell *via*bility was determined by using the alamarBlue^®^ Assay Kit (Thermo Fisher Scientific Inc.) at 72 h after treatment. At the time of detection, the medium was removed, and cells were incubated with alamarBlue^®^ reagent for 2 h at 37°C according to the manufacturer’s instruction. Plates were then analyzed by using a microplate reader (VERSAmax™ Tunable Microplate Reader, Molecular Devices) at a wavelength of 570 and 600 nm as reference.

### 2.4 Real-Time Quantitative Reverse Transcription PCR

Total RNA was extracted with genomic DNA removed by Illustra RNAspin Mini RNA Isolation Kit (GE Healthcare Life Sciences, Buckinghamshire, United Kingdom). 1 μg of DNase I-treated total RNA was reverse-transcribed using RevertAid H Minus First Strand cDNA Synthesis Kit (Life Technologies Corp.) into cDNA to use as the template for real-time PCR reaction and analysis. The real-time PCR reactions were conducted using QuantiNovaTM SYBR^®^ Green PCR Kit (QIAGEN, Hilden, Germany) on CFX Connect™ Real-Time PCR Detection System (Bio-Rad Laboratories, Inc., Hercules, CA, United States). The reaction was conducted according to the manufacturer’s instructions that an initial denaturation was at 95°C for 5 min, followed by 40 cycles of denaturing at 95°C for 5 s and combined annealing/extension at 60°C for 10 s. The primer sequences were shown as follows (Table 1): *Integrin αv*, *Integrin β3*, *Homo sapiens* cyclin D1 (*CCND1*), *c-Myc*, Vascular endothelial growth factor A (*VEGF-A*), *Homo sapiens* Caspase 2 (*CASP2*), matrix metalloproteinase-9 *(MMP-9)*, *Homo sapiens* BCL2-antagonist of cell death (*Bad*) and *Homo sapiens* programmed death-ligand 1 (*PD-L1*).

**TABLE 1:** Primer sequences for the qPCR.

	Forward	Reverse
*Integrin αv*	5′-GAT​TCC​AAA​CTG​GGA​GAC-3′	5′-AAG​GCC​ACT​GAA​GAT​GGA​GC-3′
*Integrin β3*	5′-CGA​GTG​CCT​CTG​GTC​AAT-3′	5′-AGAAGTCGTCACAC-3′
*CCND1*	5′-CAA​GGC​CTG​AAC​CTG​AGG​AG-3′	5′-GAT​CAC​TCT​GGA​GAG​GAA​GCG-3′
*c-Myc*	5′-TTC​GGG​TAG​TGG​AAA​ACC​AG-3′	5′-CAG​CAG​CTC​GAA​TTT​CTT​CC-3′
*VEGF-A*	5′-TAC​CTC​CAC​CAT​GCC​AAG​TG-3′	5′-GAT​GAT​TCT​GCC​CTC​CCT​CCT​T-3′
*CASP2*	5′-GCA​TGT​ACT​CCC​ACC​GTT​GA-3′	5′-GAC​AGG​CGG​AGC​TTC​TTG​TA-3′
*MMP-9*	5′ TGT​ACC​GCT​ATG​GTT​ACA​CTC​G-3′	5′ GGC​AGG​GAC​AGT​TGC​TTC​T-3′
*Bad*	5′-CTT​TAA​GAA​GGG​ACT​TCC​TCG​CC-3′	5′-CTT​TAA​GAA​GGG​ACT​TCC​TCG​CC-3′
*PD-L1*	5′-GTT​GAA​GGA​CCA​GCT​CTC​CC-3′	5′-ACC​CCT​GCA​TCC​TGC​AAT​TT-3′

### 2.5 Western Blotting Analysis

To examine the effects of doxycycline on the signaling pathways, Western blot analyses were conducted to quantify protein expression levels of pFAK, pERK1/2, and PD-L1 in MDA-MB-231 cells. For western blot analyses, cells were lysed and extracted protein samples were separated on 10% sodium dodecyl sulfate-polyacrylamide gel (SDS-PAGE). A 15-μg quantity of protein was loaded in each well with 5x sample buffer and the samples were separated by electrophoresis at 100 V for 2 h. The separated proteins were transferred from the polyacrylamide gel to Millipore Immobilon-PSQ Transfer PVDF membranes (Millipore, Billerica, MA, United States) with the Mini Trans-Blot^®^ Cell (Bio-Rad Laboratories, Inc.). Membranes then were incubated with NaCl/Tris blocking buffer containing 2% BSA (bovine serum albumin). Membranes were incubated with primary antibodies to phosphorylated (phospho)-ERK, phosphor-FAK, and their corresponding proteins (Cell Signaling Technology, Inc, Beverly, MA, United States), PD-L1, and GAPDH (GeneTex International Corp, Hsinchu City, Taiwan) overnight at 4°C. The proteins were detected with HRP-conjugated secondary antibodies and Immobilon™ Western HRP Substrate Luminol Reagent (Millipore). Western blots were visualized and recorded with the Amersham Imager 600 (GE Healthcare Life Sciences, Pittsburgh, PA, United States ). The densitometric analysis of Western blot was conducted by ImageJ 1.47 software (National Institute of Health, United States ) according to the software instruction.

### 2.6 Statistical Analysis

All of the collected data for immunoblot, nucleotide densities, and cell densities were analyzed by IBM^®^SPSS^®^ Statistics software version 19.0 (SPSS Inc, Chicago, IL, United States). One-way analysis of variance (ANOVA) with Duncan’s post-hoc test was conducted for multiple groups’ comparison and Student’s *t*-test was also conducted. The *p*-values < 0.05 (* or #), 0.01 (** or ##) and 0.001 (*** or ###) as the threshold for significance.

## 3 Results

### 3.1 Doxycycline Induces Anti-Proliferation in Breast Cancer Cells

**FIGURE 1:**
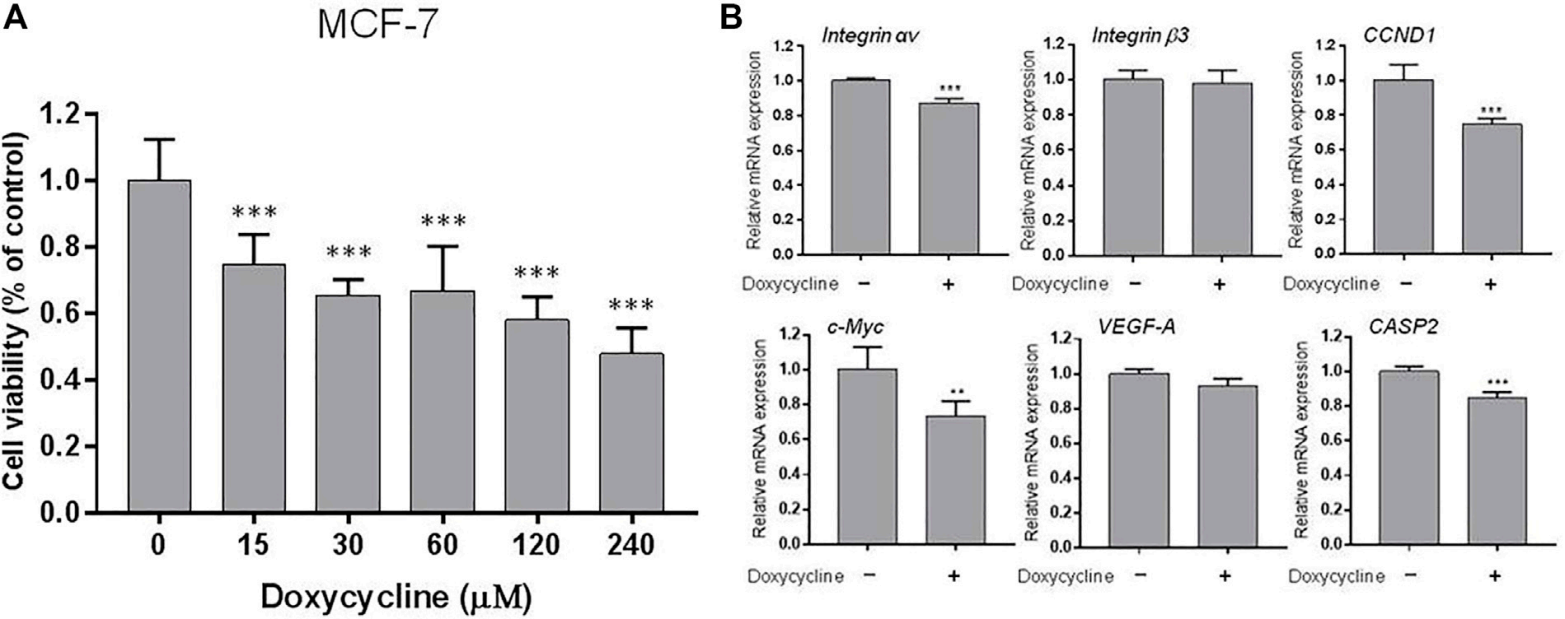
Doxycycline induces anti-proliferation and gene expression in ER-positive breast cancer cells: **(A)** MCF-7 cells were seeded in 96-well plates and treated with different concentrations of doxycycline (0, 15, 30, 60, 120, 240 µM) for 72 h. Cell *via*bility was examined with an alamarBlue^®^ assay. N = 6. ANOVA was used to assess statistical significance. Data were expressed as mean ± SD; ***p < 0.001, compared to the untreated control; **(B)** Cells were seeded in 6-well plates. Then, cells were starved for 24 h and treated with doxycycline (120 µM) for 24 h. Total RNA was extracted and qPCR analysis was performed. The number of independent experiments (*n*) = 3. A Student’s t-test was used to assess statistical significance. Data were expressed as mean ± standard de*via*tion; **p < 0.01, ***p < 0.001, compared to the untreated control.

**FIGURE 2:**
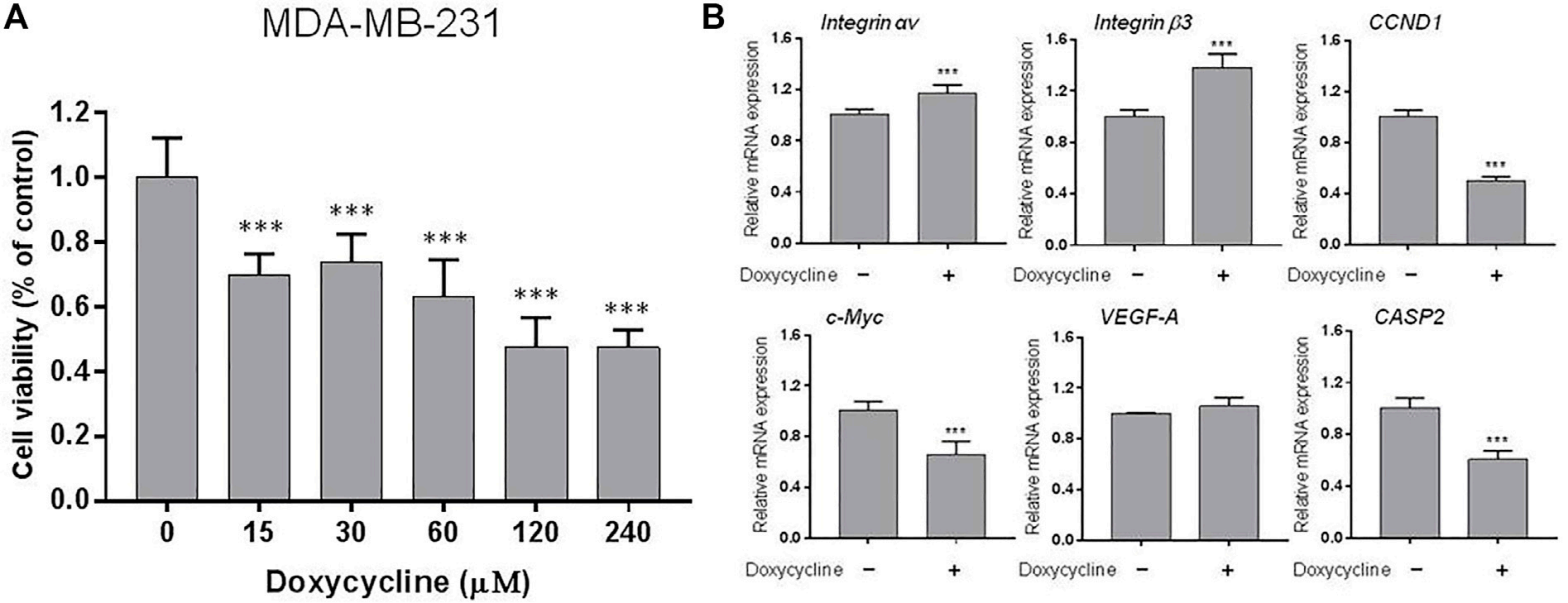
Doxycycline induces anti-proliferation and gene expression in ER-negative breast cancer cells: **(A)** MDA-MB-231 cells were seeded in 96-well plates and treated with different concentrations of doxycycline (0, 15, 30, 60, 120, 240 µM) for 72 h. Cell *via*bility was examined with an alamarBlue^®^ assay. N = 6. ANOVA was used to assess statistical significance. Data were expressed as mean ± SD; ***p < 0.001, compared to the untreated control; **(B)** Cells were seeded in 6-well plates. Then, cells were starved for 24 h and treated with doxycycline (60 µM) for 24 h. Total RNA was extracted and qPCR analysis was performed. The number of independent experiments (*n*) = 3. A Student’s t-test was used to assess statistical significance. Data are expressed as mean ± standard de*via*tion; ***p < 0.001, compared to the untreated control.

To investigate the effects of doxycycline-induced anti-proliferation in different types of breast cancer cells, ER-positive MCF-7 cells and ER-negative MDA-MB-231 cells were seeded in 96 well trays. Cells were fed with serum-free medium for 48 h and then re-fed with 10% FBS medium containing various concentrations of doxycycline. The anti-proliferative effect was detected by Alamar Blue Assay kit at the end of treatment. Results indicated that doxycycline inhibited cell proliferation in a concentration-dependent manner in both MCF-7 and MDA-MB-231 cells (Figures 1A, 2A).

Because doxycycline suppressed cell growth in both ER-positive and negative breast cancer cells, we investigated whether doxycycline could stimulate the expression of pro-apoptotic genes and inhibit proliferative genes. MCF-7 cells were harvested and total cellular RNA was extracted after 24 h treatment with doxycycline (120 μM). The qPCR was conducted for *integrin αv*, *β3*, *CCND1*, *c-Myc*, *VEGF-A*, *CASP2*. Doxycycline suppressed *integrin αv* significantly but not *integrin β3* in MCF-7 cells (Figure 1B). It also inhibited proliferative genes such as *CCND1* and *c-Myc*. In addition, doxycycline had no significant effect on the angiogenic gene *VEGF-A*. Interestingly, doxycycline also inhibited pro-apoptotic gene expression (*CASP2*).

Similar studies were performed in triple negative breast cancer MDA-MB-231 cell line. MDA-MB-231 cells were harvested and total RNA was extracted after treatment with doxycycline (60 μM) for 24 h. The qPCR was conducted for *integrin αv*, *integrin β3*, *CCND1*, *c-Myc*, *VEGF-A*, *CASP2* (Figure 2B). Doxycycline treatment inhibited the expression of *CCND1* and *c-Myc* in MDA-MB-231 cells. Instead, doxycycline treatment did not suppress the expression of *integrin αv*, *β3*, and *VEGF-A* in MDA-MB-231 cells. On the other hand, doxycycline increased the expression of *integrin αv* and *integrin β3* (Figure 2B). Moreover, doxycycline-induced gene expressions of *integrin αvβ3* displayed different patterns between MCF-7 and MDA-MB-231 cells. These results indicated that doxycycline might promote different pathways to induce anti-cancer growth in ER-positive and ER-negative breast cancer cells. The antiproliferative effect demonstrated that the cell *via*bility of ER-negative breast cancer MDA-MB-231 cells was lower than that of doxycycline-treated MCF-7 cells (Figures 1A, 2A).

### 3.2 Integrin αvβ3 Downstream ERK1/2 Plays an Important Role in Doxycycline-Induced Biological Activities in ER-Negative Breast Cancer Cells

**FIGURE 3:**
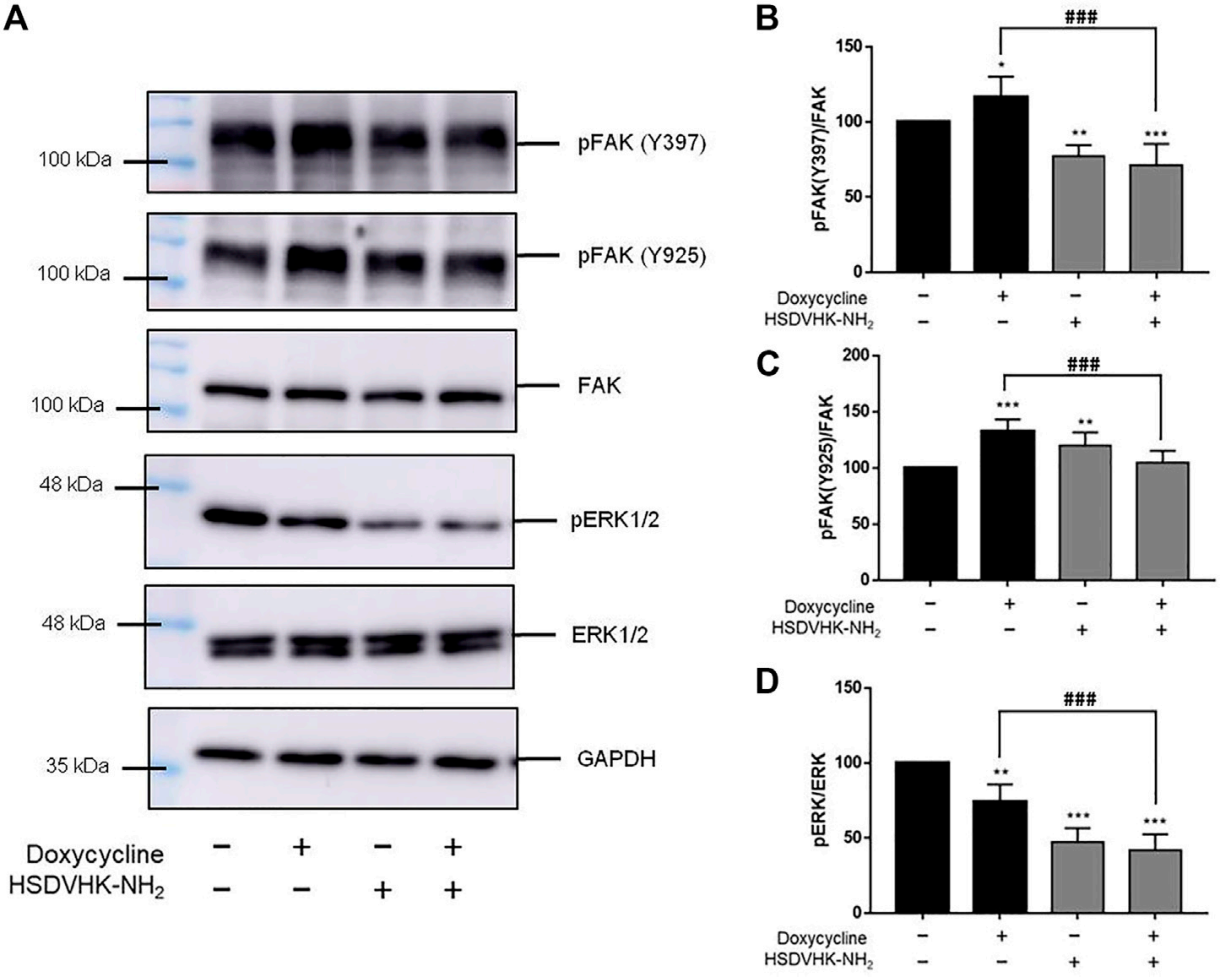
Blockage of integrin αvβ3 activity inhibits doxycycline-induced signal transduction in breast cancer MDA-MB-231 cells. **(A)** Cells were pre-treated with HSDVHK-NH_2_ (10 µM) for 24 h, then treated with doxycycline (60 µM) for 24 h. Cells were harvested and protein was extracted for Western blotting analysis of pFAK (Y397), pFAK (Y925), FAK, pERK1/2, and total ERK1/2. The HSDVHK-NH_2_ compound reduced doxycycline-induced phosphorylation of Y397 **(B)**, Y925 **(C)**, and pERK1/2 **(D)**. Numbers of independent studies (*n*) = 3. ANOVA was used to assess statistical significance. *p < 0.05, **p < 0.01, ***p < 0.001, compared to the untreated control. ###*p* < 0.001, compared to doxycycline alone.

In our previous studies, thyroid hormone binds to the receptor on integrin αvβ3 to activate signal transduction and regulate gene expression of *integrin αvβ3* (Lin et al., 2013; Hsieh et al., 2017; Abadi et al., 2021) and promote cancer cell proliferation (Lin et al., 2016b; Ho et al., 2020). Our recent findings show that doxycycline affects both gene expression of *integrin αv* and *β3* in MDA-MB-231 cells (Figure 2B). Therefore, we investigated the role of integrin αvβ3 on doxycycline-induced signal transduction in MDA-MB-231 cells by a specific integrin αvβ3 inhibitor, HSDVHK-NH_2_ (10 µM) for 24 h, followed by doxycycline (60 μM) for 24 h. Proteins were extracted and Western blotting analyses were conducted to examine FAK, phosphoFAK, and phosphoERK1/2 (Figure 3). Doxycycline induced the gene expression of *integrin αvβ3*, which then activated downstream FAK phosphorylation. The effects of doxycycline on FAK phosphorylation and *integrin αvβ3* expression were consistent in MDA-MB-231 cells. Doxycycline treatment significantly increased the phosphorylation of Tyr397/925 (Figures 3B,C). Notably, HSDVHK-NH_2_ decreased Tyr397 phosphorylation, whereas doxycycline did not affect pFAK after HSDVHK-NH_2_ pretreatment. However, the doxycycline-induced pFAK (Y397) (Figure 3B) and pFAK (Y925) (Figure 3C) were significantly reduced by the integrin αvβ3 inhibitor (HSDVHK-NH_2_). These results implied that doxycycline interacted with integrin αvβ3 and activated FAK phosphorylation. Although the active binding site might be different from that of HSDVHK-NH_2_, the activation of FAK could still be attenuated by HSDVHK-NH_2_. On the other hand, doxycycline inhibited the further downstream of integrin αvβ3, the ERK1/2 phosphorylation, and the specific mechanism of action relevant to pERK1/2 inhibition remained unclear. Regardless, HSDVHK-NH2 significantly reduced the inhibitory effect of doxycycline on ERK1/2 phosphorylation (Figure 3D).

### 3.3 Doxycycline Modulates Different Genes Expression in ER-Negative Breast Cancer Cells

**FIGURE 4:**
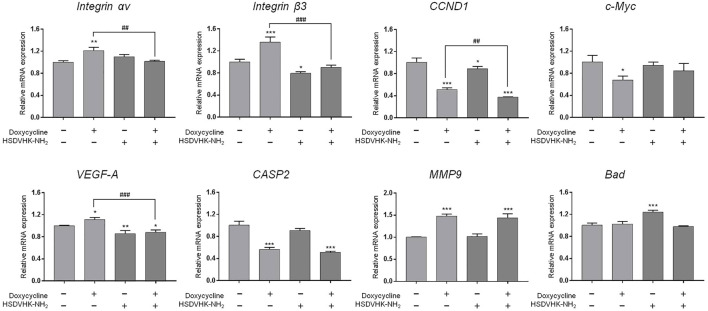
Blockage of integrin αvβ3 activity inhibits doxycycline-induced gene expression in breast cancer MDA-MB-231 cells. MDA-MB-231 cells were pre-treated with HSDVHK-NH_2_ (10 µM) for 24 h, then treated with doxycycline (60 µM) for 24 h. Cells were harvested. RNA was extracted for qPCR. Numbers of independent studies (*n*) = 3. ANOVA was used to assess statistical significance. *p < 0.05, **p < 0.01, ***p < 0.001, compared to the untreated control. ##*p* < 0.01, ###*p* < 0.001, compared to doxycycline alone.

We further investigated the effect of integrin αvβ3 on doxycycline-induced gene expression in breast cancer MDA-MB-231 cells. Cells were pre-treated with a specific integrin αvβ3 inhibitor, HSDVHK-NH_2_ (10 µM) for 24 h, followed by 60 μM doxycycline treatment for 24 h. Cells were harvested, and total RNA was extracted. The qPCR was conducted for *integrin αv*, *integrin β3*, *CCND1*, *c-Myc*, *VEGF-A*, *CASP2*, *MMP9*, and *Bad*. As shown in Figure 4, doxycycline inhibited a cell-cycle related gene (*CCND1*) and the oncogene (*c-Myc*) in MDA-MB-231 cells, whereas *integrin αv*, *integrin β3*, *VEGF-A*, *MMP-9*, and *Bad* were not suppressed. The effects of doxycycline-induced gene expression were disturbed in the presence of HSDVHK-NH_2_. Although doxycycline significantly increased the expression of integrin αv and β3 mRNA, the increased effect of gene expression could be reversed by HSDVHK-NH_2_. Results presented in Figures 3, 4 suggested that doxycycline might modulate signal transduction by regulating integrin αvβ3 in MDA-MB-231 cells. It would be interesting to investigate whether doxycycline-induced biological activity is also affected by blocking the integrin αvβ3 binding site.

**FIGURE 5:**
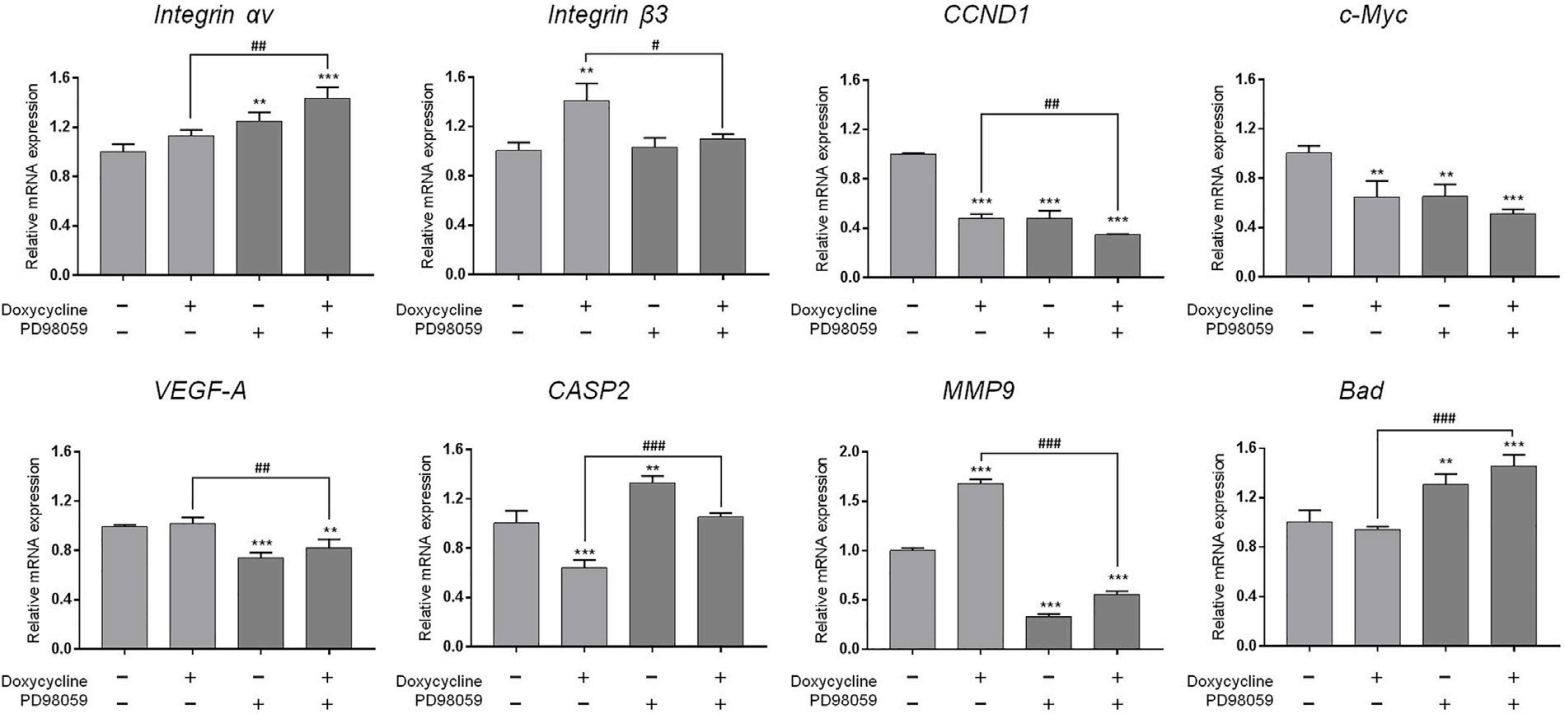
ERK1/2 activation plays an important role in doxycycline-induced gene expression in breast cancer MDA-MB-231 cells. MDA-MB-231 cells were pre-treated with PD98059 (25 µM) for 24 h, then treated with doxycycline (60 µM) for 24 h. Cells were harvested. RNA was extracted for qPCR. Numbers of independent studies (*n*) = 3. ANOVA was used to assess statistical significance. **p < 0.01, ***p < 0.001, compared to the untreated control. #*p* < 0.05, ##*p* < 0.01, ###*p* < 0.001, compared to doxycycline alone.

Since our findings (Figure 3) and others had shown that doxycycline inhibited ERK1/2 activation, we further investigated the role of the ERK1/2-dependent signal transduction pathway in doxycycline-induced biological activities in breast cancer MDA-MB-231 cells. MDA-MB-231 cells were treated with 60 µM doxycycline in the presence or absence of an ERK1/2 inhibitor, PD98059 (25 µM) for 24 h. Cells were harvested and total RNA was extracted for qPCR (Figure 5). The results are similar to Figure 4. Doxycycline inhibited *CCND1* and *c-Myc* in MDA-MB-231 cells, whereas *integrin αv*, *integrin β3*, *VEGF-A*, *MMP-9*, and *Bad* were not suppressed. These results were consistent with pre-treatment of HSDVHK-NH_2_. In addition, the gene expressions of *integrin β3*, the proliferative genes (*CCND1*), the angiogenic gene (*VEGF-A*), and the migration-related gene (*MMP9*) were further inhibited by PD98059 significantly. However, the pro-apoptotic gene (*CASP2* and *Bad*) was significantly activated by PD98059. These results indicate that activated ERK1/2 is involved in doxycycline-induced gene expressions.

### 3.4 Doxycycline Inhibits *PD-L1* Expression in ER-Negative Breast Cancer Cells

**FIGURE 6:**
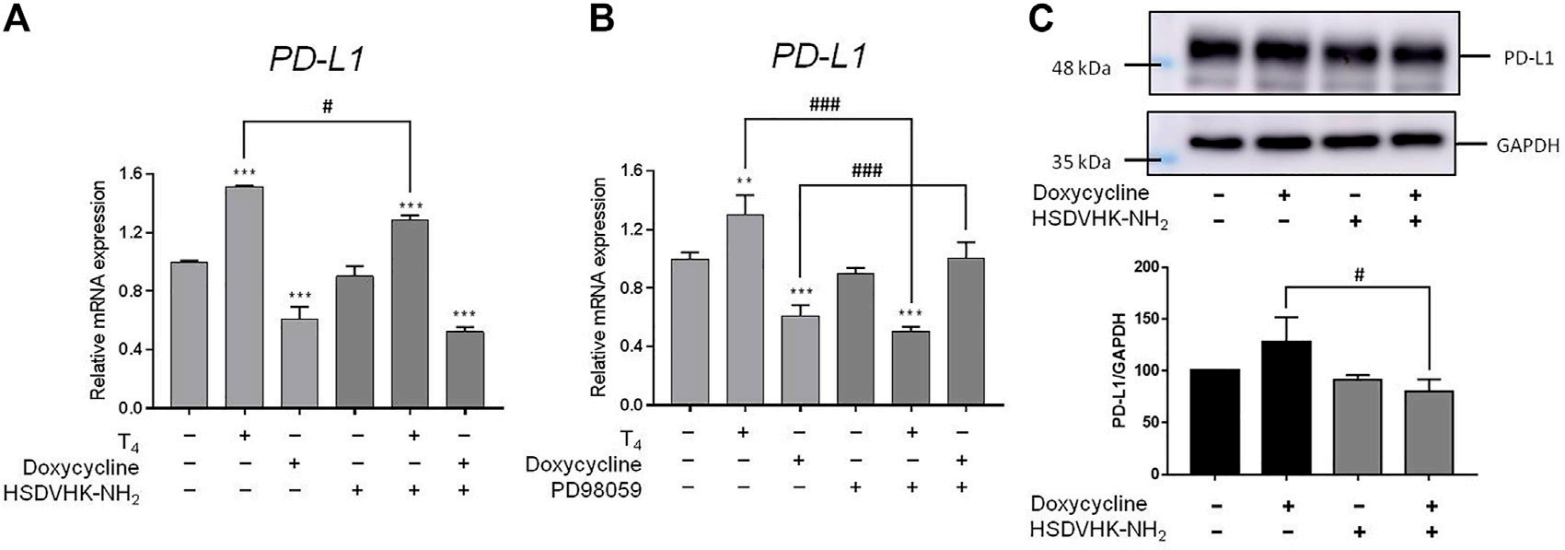
Integrin αvβ 3 and ERK1/2 collaborate to thyroxine- or doxycycline-induced *PD-L1* expression in breast cancer MDA-MB-231 cells. MDA-MB-231 cells were pre-treated with **(A)** HSDVHK-NH_2_ (10 µM) or **(B)** PD98059 (25 µM) for 24 h, then treated with T_4_ (100 nM) or doxycycline (60 µM) for 24 h. Cells were harvested. RNA was extracted for qPCR. Numbers of independent studies (*n*) = 3. ANOVA was used to assess statistical significance. **p < 0.01, ***p < 0.001, compared to the untreated control. #*p* < 0.05, ###*p* < 0.001, compared to doxycycline or T_4_ treatment alone. **(C)** MDA-MB-231 cells were pre-treated with HSDVHK-NH_2_ (10 µM) for 24 h, then treated with doxycycline (60 µM) for 24 h. Cells were harvested and protein was extracted for PD-L1 expression analysis. Numbers of independent studies (*n*) = 3. ANOVA was used to assess statistical significance. #*p* < 0.05, compared to doxycycline alone.

Activation of ERK1/2 is also involved in *PD-L1* gene expression in cancer cells (Lin et al., 2016a). Since doxycycline inhibited ERK1/2 activation, we further investigated the role of integrin αvβ3 on the inhibitory effect of *PD-L1* expression induced by doxycycline in MDA-MB-231 cells (Figure 6). Thyroxine (T_4_) was used as a positive control for integrin αvβ3 binding (Lin et al., 2016b; Davis et al., 2018; Lin et al., 2018; Davis et al., 2020; Davis et al., 2021). T_4_ increased *PD-L1* expression, and the inducible expression of *PD-L1* could be reversed by an integrin αvβ3 inhibitor (HSDVHK-NH_2_) (Figure 6A). Conversely, doxycycline suppressed *PD-L1* expression, and the levels of *PD-L1* mRNA were not significantly altered by the integrin αvβ3 inhibitor. Of note, doxycycline increased PD-L1 protein accumulation in MDA-MB cells (Figure 6C). The effect was significantly inhibited by HSDVHK-NH2 (Figure 6C). These results suggested that integrin αvβ3 may play different regulations on *PD-L1* expression and its protein accumulation. To determine the role of ERK1/2 in doxycycline-induced *PD-L1* expression, MDA-MB-231 cells were pre-treated with an ERK1/2 inhibitor (PD98059) before treatment with doxycycline or thyroxine. T_4_-induced *PD-L1* expression was significantly reduced in the presence of PD98059 (Figure 6B). On the other hand, suppressive effect of doxycycline on *PD-L1* expression was interrupted by PD98059, therefore, the suppressive expression of *PD-L1* might be up-regulated by negative feedback. These results indicate that regulations of *PD-L1* expression by T_4_ and doxycycline were ERK1/2-dependent.

In summary, both doxycycline and thyroxine interacted with integrin αvβ3 and regulated ERK1/2 activation consequently modulating proliferation in ER-negative breast cancer MDA-MB-231 cells. Eventually, doxycycline inhibited ERK1/2 activation, a downstream of integrin αvβ3, regulated gene expression and suppressed cancer cell growth sequentially in ER-negative MDA-MB-231 cells.

## 4 Discussion

Several studies have shown that doxycycline can induce apoptosis in a range of cancer cells (Mouratidis et al., 2007; Son et al., 2009; Dijk et al., 2020; Alsaadi et al., 2021), indicating that the drug can be applied as an anti-cancer drug for cancer patients in clinical practice. In addition, doxycycline can reduce cancer stem cell (CSC) progression through apoptosis (Yang et al., 2015; Matsumoto et al., 2017), and it has been tested in the clinical trial study to reduce cancer stem cells (CSCs) in breast cancer patients *in vivo* (Scatena et al., 2018). Evidence has supported that breast cancers originate from breast cancer stem cells (BCSCs) (Zhang et al., 2017). The study also indicates that doxycycline inhibits the vitality and growth of BCSCs by reducing effective mammosphere formation, migration, and infiltration. Biosynthesis in mitochondria plays a vital role in clonal proliferation and survival independent of CSC scaffolds. Therefore, it makes mitochondria to be an important target for new treatment methods. Doxycycline impairs mitochondrial function and protects hypoxia-induced apoptosis in glioma cells (Luger et al., 2018). It also shows that doxycycline targets mitochondria (De Luca et al., 2015; Dijk et al., 2020). Besides, doxycycline inhibits NF-κB activation and reduces plasma lysophosphatidate concentrations to attenuate breast cancer-related inflammation (Tang et al., 2017). In our study, doxycycline was shown to inhibit the proliferation of ER-positive and negative breast cancer cells in a concentration-dependent manner (Figures 1, 2). Studies by Le Zhang et al. indicate that doxycycline suppressed the transcription of autophagy markers protein 1 light chain 3-B (LC-3B) I and LC-3BII (Zhang et al., 2017). Doxycycline treatment effectively inhibits epithelial-to-mesenchymal transition (EMT) and cancer rod-like features in breast cancer cells. In addition, doxycycline has been shown to significantly down-regulate transcription of the stem cell factors such as octamer-binding transcription factor 4 (Oct4), sex determining region Y-Box transcription Factor 2 (Sox2), Nanog, and CD44 (Zhang et al., 2017). Those observations suggested that autophagy inhibited by doxycycline may be in part due to the effects on expression of stem cell markers, EMT, and proliferation (Zhang et al., 2017).

Doxycycline is verified to block cancer cell progression by different mechanisms (Sagar et al., 2010; Hadjimichael et al., 2020; Yi et al., 2021). The previous studies have shown that doxycycline suppresses MMP expression and FAK phosphorylation to inhibit leukemic cell migration (Wang et al., 2015). Accordingly, doxycycline inhibits migration and metastasis in cancer cells that may be related to the activities of integrins (Sun et al., 2009). However, the study of integrin αvβ3 regulating breast cancer growth through doxycycline is unclear. In the present study, our preliminary gene screen results indicated that doxycycline down-regulated expression of *integrin αv* and the proliferative genes (*CCND1* and *c-Myc*) and the pro-apoptotic gene (*CASPS2*) in MCF-7 cells (Figure 1B). Interestingly, doxycycline treatment did not suppress the expression of *integrin αv* in MDA-MB-231 cells (Figure 2B). Alternatively, doxycycline increased expression of *integrin αv* and *integrin β3*, but inhibited the expression of *CCND1*, *c-Myc* and *CASPS2* in MDA-MB-231 cells (Figure 2B).

The integrin αvβ3 is overexpressed in endothelial cells and interrupting ligand binding to block integrin αvβ3 function may produce anti-angiogenic effects (Weis and Cheresh, 2011). Expression of integrin αvβ3 on different cell types contributes to cell growth and mobility. Although integrin αvβ3 may also express on normal cells, the tertiary conformation of integrin αvβ3 on cancer cells attract potential antagonists to interrupt multiple aspects of cancer progression. Our results indicate that doxycycline inhibits expression of *integrin αv* in MCF-7 cells but not in MDA-MB-231 cells (Figures 1B, 2B). Interestingly, our results showed that the half-maximal inhibitory concentration (IC_50_) of MDA-MB-231 cells was between 60 and 120 μM (Figure 2A), whereas the IC_50_ of MCF-7 cells was as high as 120–240 μM (Figure 1A). These results imply that MDA-MB-231 cells are more sensitive to doxycycline than MCF-7 cells. A previous study showed that integrin αvβ3 was overexpressed in MDA-MB-231 cells compared to MCF-7 cells (Godugu et al., 2021). The higher αvβ3 expression was found in MDA-MB-231 (21%) and the lower expression of αvβ3 was found in the MCF-7 breast cancer cell line (9%) by using flow cytometry. Accordingly, we support that doxycycline-induced antiproliferation may be related to integrin αvβ3 activity in ER-negative breast cancer MDA-MB-231 cells. On the other hand, anti-β1-integrin antibodies showed inhibitory effects on leukemic cell migration induced by doxycycline (Wang et al., 2015) suggesting that doxycycline may inhibit cancer migration *via* integrin functional inhibition. As shown in Figure 3, doxycycline significantly activates both Tyr397/925 phosphorylation of FAK, but inhibits the ERK1/2 phosphorylation. The results are partially consistent with the previous study showing that doxycycline highly inhibits the levels of ERK/MAPK proteins and their regulated transcriptional activity (Pandian et al., 2020). Furthermore, we investigate whether the FAK and ERK1/2 phosphorylation are suppressed in the presence of an integrin αvβ3 inhibitor (HSDVHK-NH_2_). Our findings demonstrate that both FAK and ERK1/2 activities induced by doxycycline are significantly inhibited by HSDVHK-NH_2_. In addition, the combination with an integrin αvβ3 inhibitor enhances the inhibition of ERK1/2 phosphorylation compared to doxycycline alone (Figure 3D). Previously, we have investigated that thyroxine (T_4_) can induce the ERK1/2 phosphorylation and PD-L1 protein accumulation (Lin et al., 2016a). Compared with our previous study, the results suggest that both thyroxine and doxycycline can interact with integrin αvβ3 to regulate ERK1/2 signal transduction pathways. The former activates ERK1/2 and the latter suppresses ERK1/2 activation. Besides, the effects of doxycycline-induced gene expression are also disturbed in the presence of HSDVHK-NH_2_ (Figure 4). We further investigated whether ERK1/2 inhibition affects doxycycline-induced gene expressions. Effects of doxycycline-induced gene expression are disturbed in the presence of a MEK inhibitor (PD98059) in MDA-MB-231 cells (Figure 5). These results also provide evidence that doxycycline interacts with integrin αvβ3 to regulate various gene expressions *via* ERK1/2 signaling and suppress cancer growth in ER-negative breast cancer cells.

In our previous study, the activation of *PD-L1* expression is up-regulated by ERK1/2-dependent phosphorylation (Lin et al., 2016a). Thyroxine (T_4_) acts on integrin αvβ3 to activate specific signal transduction. T_4_ induces ERK1/2 activation and cell proliferation *via* binding to cell surface integrin αvβ3 (Lin et al., 2016b; Davis et al., 2018; Lin et al., 2018; Davis et al., 2020; Davis et al., 2021). Blockage of integrin αvβ3 signaling and ERK1/2 pathway (Figures 6A,B) reduces the biological activities of thyroxine by an integrin αvβ3 inhibitor (HSDVHK-NH_2_) or a MEK inhibitor (PD98059). Those results confirm our previous studies that thyroxine *via* integrin αvβ3 activates the ERK1/2-dependent signal transduction pathway to modulate proliferation in cancer cells (Lin et al., 2013; Davis et al., 2014; Davis et al., 2015; Lin et al., 2016a; Lin et al., 2016b). In contrast with T_4_, doxycycline down-regulates the gene expression of *PD-L1*, and the suppressive effect is partially removed by a MEK blocker, PD98059 (Figure 6B). Those results illustrate that doxycycline may modulate the *PD-L1* expression *via* the ERK1/2 pathway.

**FIGURE 7:**
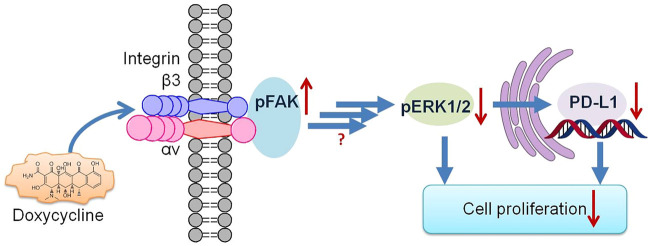
The signaling pathways of doxycycline-induced anti-proliferation in human breast cancer MDA-MB-231 cells.

In conclusion, doxycycline is a widely used antibiotic for a long time, therefore, it is safer than other novel drugs and has well-established pharmacokinetics. It is merit to investigate for other applications such as anticancer. The summary of doxycycline-induced antiproliferative effect on breast cancer cells is illustrated in Figure 7. Our findings indicated that doxycycline exhibits dose-response regarding anti-proliferation in ER-positive (MCF-7) and ER-negative (MDA-MB-231) breast cancer cells. Results show that ER-negative cells are more sensitive to the anticancer activities of αvβ3 integrin and doxycycline. Doxycycline modulates different expression patterns of genes in those types of breast cancer cells. Furthermore, doxycycline inhibits ERK1/2 phosphorylation, a downstream of integrin αvβ3, and suppresses PD-L1 expression. Blockade of PD-1/PD-L1 should prevent cancer cells from escaping immune defenses, thereby enhancing doxycycline-induced antitumor activity, ultimately resulting in a synergistic therapeutic effect. Collectively, these findings suggest that doxycycline interacts with integrin avβ3 to downregulate ERK1/2 phosphorylation and downstream *PD-L1* signaling and gene expression, thereby inhibiting cancer cell growth.

## Data Availability

The original contributions presented in the study are included in the article/supplementary material, further inquiries can be directed to the corresponding author.
